# Myocardial extracellular volume fraction with spectral detector computed tomography for risk stratification in non-ischemic heart failure

**DOI:** 10.1007/s11547-025-02002-1

**Published:** 2025-04-30

**Authors:** Jie Deng, Yu Wang, Tianfu Qi, Zhiming Li, Hongen Zheng, Yan Wu, Lin Lu, Deyan Li, Dan Han, Wei Chen

**Affiliations:** 1 Department of Radiology, First Affiliated Hospital of Kunming Medical University, 295 Xichang Road, Kunming, 650032 China; 2 Department of Nuclear Medicine, Sun Yat-Sen Memorial Hospital, No. 107, The West of Yanjiang Road, Guangzhou, 510120 China; 3 Department of Ultrasound, First Affiliated Hospital of Kunming Medical University, 295 Xichang Road, Kunming, 650032 China

**Keywords:** Extracellular volume fraction, Late iodine enhancement, Non-ischemic heart failure, Spectral detector computed tomography

## Abstract

**Purpose:**

To validate the feasibility of using late iodine enhancement (LIE)-derived ECV on iodine density images using spectral detector computed tomography (SDCT; CT-ECV) and to assess the potential of CT-ECV for risk stratification among patients with non-ischemic heart failure (NIHF).

**Materials and methods:**

Forty-five subjects who underwent cardiac SDCT and CMR were included in the validation group to calculate and compare CT-ECV with CMR-ECV to validate CT-ECV feasibility. Another 117 subjects (82 patients with NIHF, 35 controls) who underwent SDCT were included in the experimental group to explore the potential of CT-ECV for risk stratification. ECV was measured via iodine density images and CMR T1 mapping in accordance with American Heart Association 16-segment models.

**Results:**

In the validation group, there was no significant difference between CT-ECV and CMR-ECV (*P* = 0.293), with an insignificant bias. In the experimental group, CT-ECV in patients with NIHF was significantly higher than in controls (*P* < 0.05). In 82 patients with NIHF, CT-ECV in HFrEF ( HF with reduced ejection fraction: LVEF ≤ 40%) patients was statistically higher than that of HFmEF (HF with mildly reduced ejection fraction: LVEF 41–49%) and HFpEF (HF with preserved ejection fraction: LVEF ≥ 50%) patients (*P* < 0.05) and a significant difference among patients with NIHF with varied New York Heart Association classes (all *P* < 0.05); In addition, Kaplan–Meier survival curves and Log-rank test demonstrated that NIHF patients with CT-ECV ≥ 31.29% had higher probability of MACE than NIHF patients with CT-ECV < 31.29% (*P* < 0.001).

**Conclusion:**

LIE-derived CT-ECV on iodine density images using SDCT is a promising practical alternative to CMR-ECV, with the potential to assist with risk stratification among patients with NIHF.

**Graphical abstract:**

In the assessment of non-ischemic heart failure, inclusion of late iodine enhancement to obtain ECV in CCTA examination may play a valuable role in future risk assessment in this clinical setting.
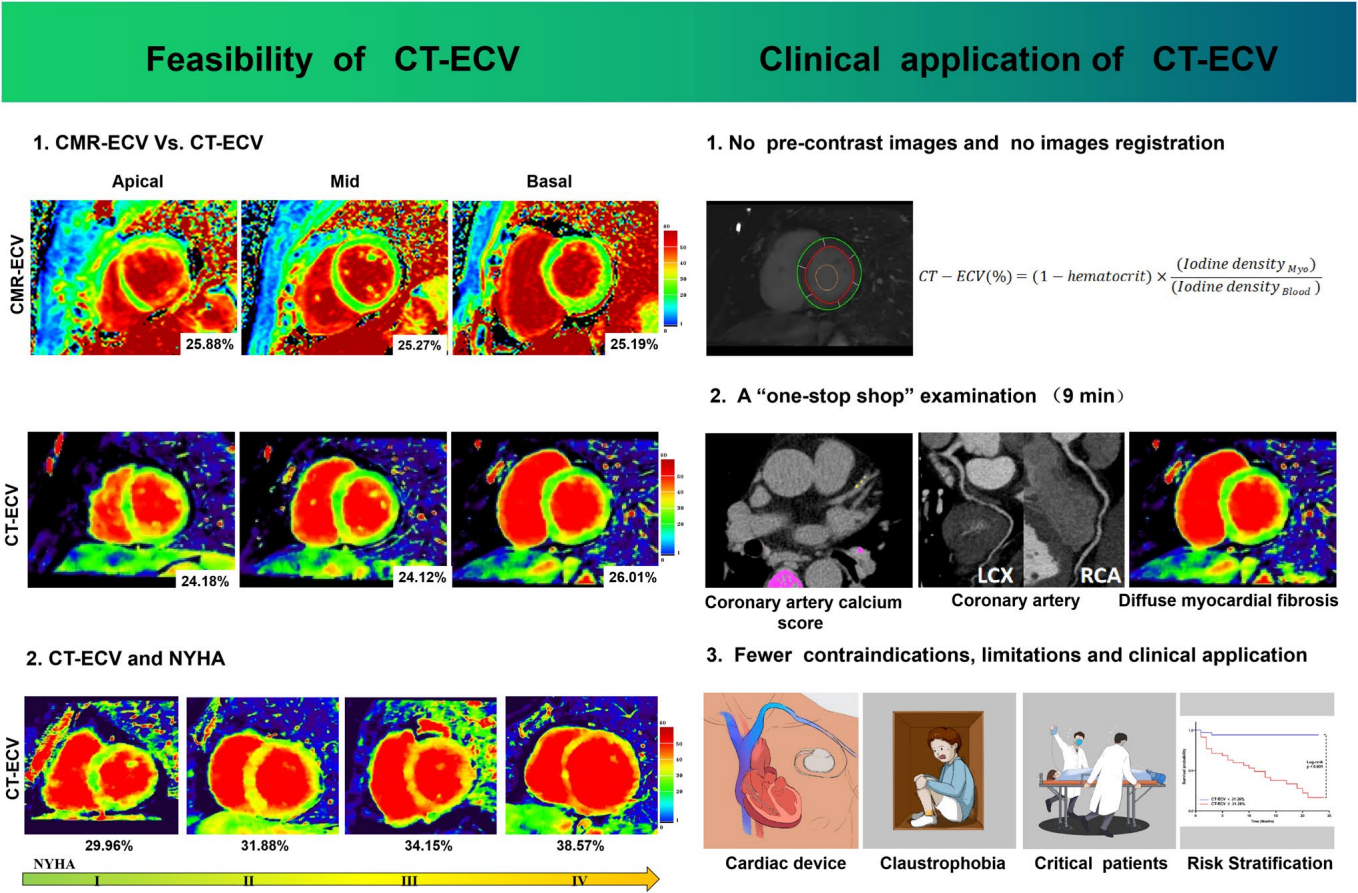

**Supplementary Information:**

The online version contains supplementary material available at 10.1007/s11547-025-02002-1.

## Introduction

Diffuse myocardial fibrosis, characterized by excess collagen deposition and expansion of the extracellular matrix, plays a crucial role in the progression of various cardiomyopathies and heart failure (HF) [1, 2]. However, its onset and progression are often overlooked, especially in patients with non-ischemic heart failure (NIHF). To address this, myocardial extracellular volume fraction (ECV) derived from cardiac magnetic resonance (CMR) T1 mapping has emerged as a noninvasive imaging biomarker for assessing and quantifying diffuse myocardial fibrosis [3, 4]. However, CMR is not widely available and has certain limitations and contraindications [5].

Computed tomography (CT) has some advantages with regard to compensating for the lack of CMR. In addition, due to its pharmacokinetics (which are shared by gadolinium and iodine contrast agents), late iodine enhancement (LIE) has gradually been accepted for its potential and promise for quantifying ECV [5–10]. The emerging dual-layer spectral detector computed tomography (SDCT) technology has the following advantages, which can improve upon the limitations of current studies involving LIE-derived CT-ECV: (1) SDCT permits the simultaneous acquisition of both low- and high-kVp data sets to generate spectral base images (SBIs). SBIs can be used to reconstruct iodine density images, which enable the direct quantification of iodine concentration in organs with high tissue resolution. This process avoids the sophisticated registration that occurs between pre- and post-contrast images. (2) Compared to other multi-detector CT modalities that require the prospective selection of the appropriate dual-energy mode before scanning to obtain iodine density images, SBIs obtained with dual-layer technology can be applied to all patients, provided that specific scan parameters are set (a tube voltage of 120 kV) during the scanning process. This approach facilitates the standardization of disease detection in routine clinical practice [11, 12]. In clinical contexts, CCTA is recommended for patients with HF who are at moderate-to-low risk of CAD, especially when new-onset HF with a possible relation to CAD is suspected, to evaluate the coronary arteries for the detection or exclusion of ischemia or NIHF. Furthermore, CCTA is useful for excluding coronary artery disease in patients with suspected non-ischemic cardiomyopathy [13]. Thus, it will be of great significance to develop a protocol that can simultaneously evaluate both the coronary arteries and diffuse myocardial fibrosis in patients with known or suspected HF in a “one-stop shop” examination.

With this in mind, our study will address the following two research questions: (1) Can LIE-derived ECV on iodine density images using SDCT quantify diffuse myocardial fibrosis in a way that is similar to CMR-ECV? (2) If so, what is the potential of CT-ECV for risk stratification among patients with NIHF?

## Methods

### Study population

The prospective study was approved by the research ethics committee of the First Affiliated Hospital of Kunming Medical University (Approval No. 2022L232). All study participants provided written informed consent before their examinations. Patients with known or suspected heart disease underwent cardiac SDCT examinations at our institution between December 2020 and March 2023.

A validation group (G_V_), which included patients who underwent both cardiac SDCT and CMR examinations within 1 week, was set up to validate the feasibility of CT-ECV via head-to-head comparison with CMR-ECV. After CT-ECV was found to be feasible, the experimental group (G_e_)—which included patients with NIHF and controls—was set up to evaluate the potential of CT-ECV for risk stratification among patients with NIHF.

The inclusion criteria for patients with NIHF were heart failure attributed to non-coronary artery disease. Non-coronary artery disease is defined as follows [14]: (1) absence of significant coronary artery disease, confirmed by coronary angiography or CCTA with a diameter stenosis < 50%; (2) no history of previous coronary intervention therapy or myocardial infarction [13]. The severity of luminal diameter stenosis is graded as follows [14]: No visible stenosis (0%), minimal stenosis (1–24%), mild stenosis (25–49%), moderate stenosis (50–69%), severe stenosis (70–99%), occluded (100%); overall amount of coronary plaque assessed using coronary artery calcium score calculation: Mild (1–100), Moderate (101–300), Severe (301–999), Extensive (> 1000).

Criteria for HF followed the 2021 European Society of Cardiology Heart Failure Guidelines [15], which include (1) symptoms and/or signs of HF (New York Heart Association [NYHA] Class ≥ I); (2) objective evidence of cardiac structural and/or functional abnormalities on echocardiography; and (3) raised B-type natriuretic peptide (BNP) ≥ 35 ng/L or N-terminal pro-BNP (NT-proBNP) ≥ 125 ng/L). In this study, the term *patients with NIHF* encompasses various subgroups of patients, such as those with dilated cardiomyopathy, hypertrophic cardiomyopathy, hypertensive heart disease, or myocarditis. These diseases were diagnosed in accordance with the relevant guidelines [16–20]. Patients with infiltrative myocardial diseases, such as cardiac amyloidosis, hemochromatosis, glycogen storage diseases, and Fabry disease, were excluded due to their distinct pathophysiological mechanisms.

The classification of patients with NIHF were first divided into three categories [15]: heart failure with reduced ejection fraction (HFrEF), mildly reduced ejection fraction (HFmrEF), and preserved ejection fraction (HFpEF). HFrEF is defined as (1) symptoms ± signs and (2) LVEF < 40%. HFmrEF includes (1) symptoms ± signs and (2) LVEF 41–49%. HFpEF is characterized by (1) symptoms ± signs, (2) LVEF ≥ 50%, and (3) objective evidence of structural or functional cardiac abnormalities consistent with LV diastolic dysfunction or elevated LV filling pressures, including raised natriuretic peptides. In addition, per the NYHA Classification system, we also divided the patients with NIHF into four subgroups: NYHA Classes I, II, III, and IV. All clinical examinations and laboratory tests, including hematocrit (HCT) values obtained from routine blood sampling, were performed within 2–4 days before or after the cardiac SDCT or CMR examination. Patients who underwent CMR and/or cardiac SDCT for chest pain, chest tightness, and palpitations but who had no abnormalities found with CMR, cardiac SDCT, electrocardiography, or myocardial enzyme testing and who had no history of alcoholism, hypertension, diabetes, drug use, or systemic disease served as controls. The key exclusion criteria for this study were as follows: contraindications for cardiac magnetic resonance (CMR) or cardiac SDCT, chronic renal failure with a current estimated glomerular filtration rate of less than 30 ml/min/1.73 m^2^, inability to provide informed consent, intolerance to examination, and age under 18.

### Clinical follow-up

Electronic and medical records were reviewed for the collection of follow-up data and/or the patient was contacted. The primary clinical endpoint was the first occurrence of a major adverse cardiovascular events (MACE), which included hospital admission for heart failure, cardiovascular mortality and all-cause mortality [21].

### Cardiac CT image acquisition

All cardiac CT images were obtained using a dual-layer spectral detector CT scanner (iQon Spectral Detector CT; Philips Healthcare, Best, The Netherlands). The cardiac SDCT scanning protocol in this study consisted of three acquisitions: (1) pre-contrast images for coronary artery calcium score calculation; (2) CCTA images for coronary artery evaluation; and (3) LIE images for diffuse myocardial fibrosis assessment. Table 1 shows the scanning parameters used in our cardiac SDCT protocol.

**Table 1:** The acquisition parameters of the cardiac computed tomography protocol

	Pre-contrast images	CCTA images	LIE images
Tube voltage (kV)	120	120	120
Effective mAs (mA)	42	567	100
Rotation time (s)	0.27	0.27	0.27
Collimation (mm)	64 × 0.625	64 × 0.625	64 × 0.625
Display field of view (mm^2^)	400	400	400
Matrix	512 × 512	512 × 512	512 × 512
Slice thickness (mm)	0.8	0.9	0.8
Slice gap (mm)	0.6	0.45	0.2

CCTA, Coronary computed tomography angiography; LIE, late iodine enhancement

Cardiac SDCT scanning protocol (Supplementary Figure 1) are briefly described as follows. First, pre-contrast images were acquired using prospective electrocardiogram (ECG)-triggered axial scans. Next, CCTA was performed using retrospective ECG-triggered axial scanning after the intravenous injection of 0.9 ml/kg of iopamidol (370 mg iodine/ml; Bracco Sine Pharma, Shanghai, China) at a flow rate of 4.5–5.0 ml/s through the right antecubital vein, followed by a 25- to 30-ml saline flush at a rate of 4.5–5.0 ml/s.

After CCTA, the additional continuous infusion of 30 ml of contrast agent for 60 s at a flow rate of 0.5 ml/s was immediately performed. Finally, late iodine enhancement (LIE) images were acquired using prospective ECG-triggered axial scans 7 min after contrast administration. The scan was conducted during the mid-diastolic phase, corresponding to 78% of the R-R interval with a ± 3% buffer. During the entire scanning process, the maximum dose of the contrast agent was kept below 100 ml [7]. Oral beta-blockers (Betaloc ZOK, 47.5 mg; AstraZeneca AB, Sweden) was administered 30 min before CT if the patient had a heart rate greater than 70 beats per minute and had not been routinely prescribed beta-blockers. All patients without contraindications received sublingual administration of nitroglycerin (Isosorbide Dinitrate Tablets, 5 mg; Jiangsu Changjiang Pharmaceutical, Jiangsu, China) 5 min before scan.

The effective radiation dose was calculated as follows: dose-length product × conversion factor of 0.014 [22].

### CMR image acquisition

All CMR images were acquired from a 1.5-T MR scanner (MAGNETOM Amira, Siemens Medical Systems, Munich, Germany) with an 18-channel cardiac coil. T1 mapping was integrated into our routine clinical imaging protocol including cine steady-state free precession (SSFP) images of the entire heart in short and long axes and late gadolinium enhancement using phase sensitive inversion recovery (PSIR). T1 mapping was acquired on the basal, mid, and apical short-axis layers of the LV myocardium before and 10 min after the administration of gadobutrol (0.15 mmol/kg Gadovist; Bayer Healthcare, Leverkusen, Germany, Germany) using an ECG-gated steady-state free-precession–based modified Look-Locker inversion recovery technique with a 5(3)3 scheme and a 4(1)3(1)2 scheme, respectively (Supplementary Table 1).

### ECV derived from CMR T1 mapping and iodine density images

To extract myocardial T1 values to calculate the CMR-ECV, the short axial layers of the LV myocardium were divided into six (basal and mid) or four (apical) segments in accordance with the American Heart Association 16-segment model. Regions of interest (ROIs) were drawn manually on each LV myocardial segment on native and post-contrast T1 maps with dedicated software (CVI42; Circle Cardiovascular Imaging, Calgary, Alberta, Canada) [23].

CT-ECV was measured on the client image viewing system (Spectral CT Viewer, Intellispace Portal; Philips Healthcare, Best, The Netherlands). The iodine density images were reconstructed from LIE scans at a specific cardiac phase, specifically at 78% of the cardiac cycle. Additionally, iodine density images reconstructed at 8-mm slice thickness and 2-mm slice gap on the basal, mid, and apical short-axis planes of the LV myocardium and in correspondence with the CMR T1 mapping.

**Fig. 1:**
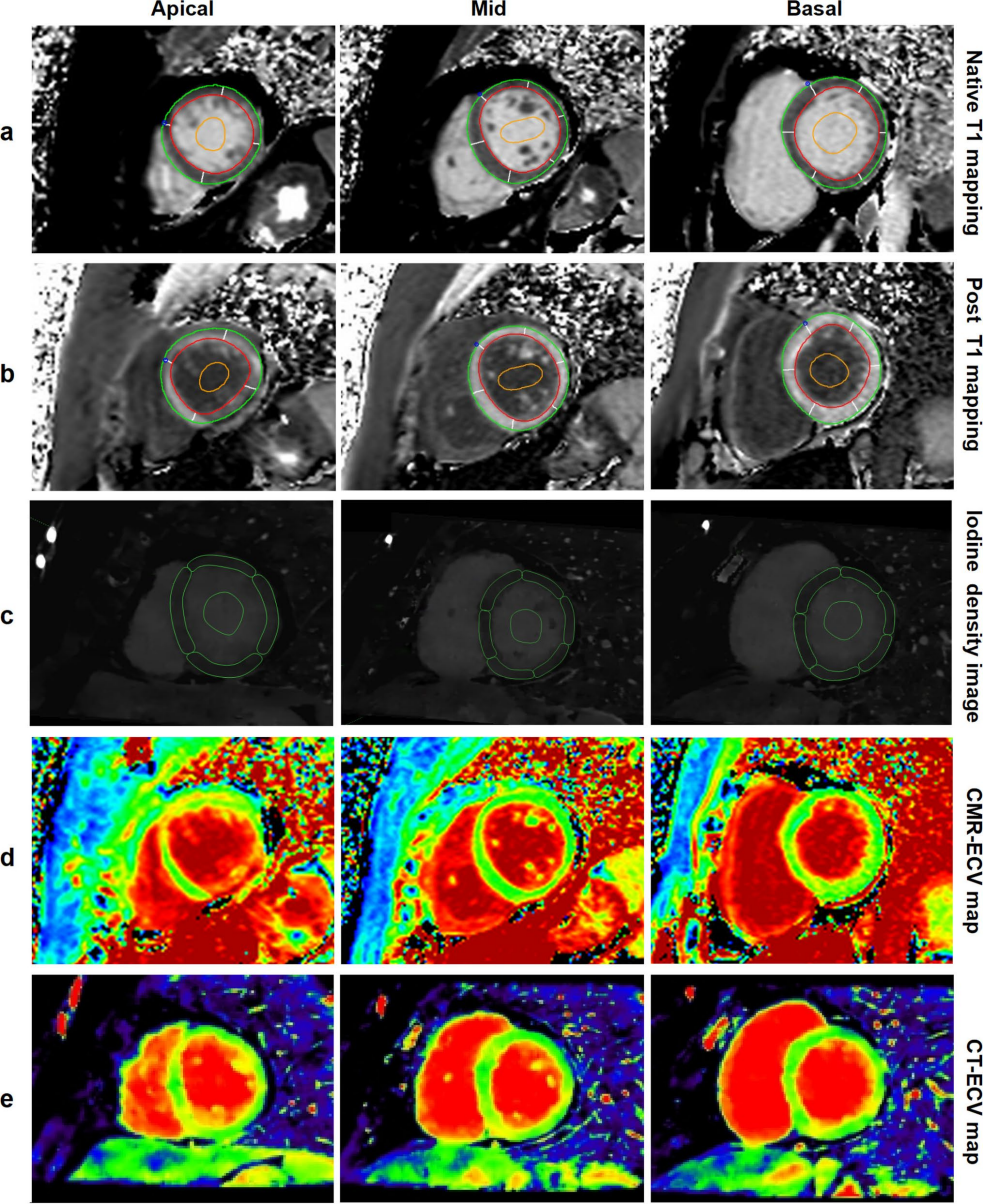
Regions of interest drawn on T1 mapping and iodine density images. **a–c** Regions of interest were manually drawn on left ventricular (LV) myocardium images from a control subject according to the American Heart Association’s 16-segment model and on the LV blood pool to calculate the extracellular volume fraction (ECV). Red outline = endocardium, green outline = epicardium, orange outline = blood pool. **a** Native T1 mapping: Native T1 values at the basal, mid, and apical layers of LV myocardium were 1072 ms, 1059 ms, and 1055 ms, respectively. **b** Post-contrast T1 mapping: Post-contrast T1 values at the basal, mid, and apical layers of LV myocardium were 427 ms, 406 ms, and 407 ms, respectively. **c** Iodine density images: Iodine density values at the basal, mid, and apical layers of LV myocardium were 0.88 mg/ml, 0.82 mg/ml, and 0.81 mg/ml, respectively. **d** CMR-ECV mapping: CMR-ECV at the basal, mid, and apical layers were 25.19%, 25.27%, and 25.88%, respectively. **e** CT-ECV mapping: CT-ECV at the basal, mid, and apical layers were 26.01%, 24.12%, and 24.18%, respectively. The global CMR-ECV and CT-ECV values were 25.45% and 24.77%, respectively

Next, to extract myocardial iodine density values, the contours of the ROIs of each segment of the LV myocardium and the blood pool on the iodine density images were matched with that of the CMR T1 mapping (Fig. 1). The segments affected by significant artifacts or poor myocardium definition were excluded to avoid distorting the measured values. All the CMR and iodine density images were analyzed by one radiologist (W.C.) with 10 years of experience in cardiac imaging diagnosis, who was blinded to the clinical data.

The endocardial and epicardial contours in T1 mapping and Iodine density images were manually drawn slice by slice by two researchers (J.D. and H.E.Z.) with 1–3 years of experience in cardiac imaging diagnosis.

The ECV values derived from CMR T1 mapping and iodine density images were calculated using the following formulas [10]:

$$\begin{aligned} & {\text{CMR}} - {\text{ECV}}\left( \% \right) = \left( {{1}/{\text{T1}}_{{\text{myo post}}} {-}{ 1}/{\text{T1}}_{{\text{myo pre}}} } \right)/\left( {{1}/{\text{T1}}_{{\text{blood post}}} {-}{ 1}/{\text{T1}}_{{\text{blood pre}}} } \right) \times \left( {{1 }{-}{\text{ hematocrit}}} \right) \\ & {\text{CT}} - {\text{ECV}}\left( \% \right) = \left( {{\text{Iodine density}}_{{{\text{myo}}}} /{\text{Iodine density}}_{{{\text{blood}}}} } \right) \times \left( {{1 }{-}{\text{ hematocrit}}} \right) \\ \end{aligned}$$

### Echocardiography for LVEF in patients with NIHF and controls

All patients underwent transthoracic echocardiography (TTE) within 2 to 4 days before cardiac SDCT or CMR examination. Transthoracic echocardiography (TTE) was performed with ultrasound equipment (EPIQ7c, Philips Medical Systems, Best, The Netherlands) and the M5S transducer (frequency range 1.5–5.0 MHz). Cardiac structural, diastolic and systolic parameters were measured including LV end-diastolic diameter (LVEDD) and LV end-systolic diameter (LVESD); LV end-diastolic volume (LVEDV) and LV end-systolic volume (LVESV); LV ejection fraction (LVEF); left atrium volume (LAV); pulmonary artery systolic pressure (PASP), E/A ratio, E/e' ratio, E', tricuspid regurgitation (TR) peak velocity and aortic valve peak gradient (mmHg). The TTE examination protocols and the analysis of the aforementioned cardiac parameters were conducted in accordance with the established guidelines [24]. The TTE was performed by an experienced cardiologist (Y.W.) with over 20 years of expertise in echocardiographic diagnosis.

### Statistical analysis

Analysis was performed using SPSS version 23 (IBM, Armonk, New York), GraphPad Prism version 8.0 (GraphPad Software, La Jolla, California), and *P* < 0.05 was defined as statistically significant.

The normality of data distribution was assessed using the Shapiro–Wilk test. Continuous variables were expressed as mean ± standard deviation (Mean ± SD) or as median and interquartile range, depending on whether the data followed a normal distribution. Categorical variables were presented as counts and percentages. The baseline characteristics between groups were compared using the Mann–Whitney U test and the Fisher exact test. CT-ECV and CMR-ECV were compared using the paired t-test. The difference in CT-ECV among groups was assessed with one-way analysis of variance and the unpaired t-test. The correlation between variables was evaluated with Pearson correlation analysis for parametric data or Spearman correlation analysis for non-parametric data.

The inter- and intra-observer variability was assessed with the intra-class correlation coefficient and the Bland–Altman plot. Bland–Altman analysis was performed to analyze biases and limits of agreement. For intra-observer variability, 20 randomly selected studies from all subjects were re-analyzed by an observer (J.D.) after a 3-month washout period. For inter-observer variability, a second observer (H.E.Z.) independently analyzed the same 20 studies, blinded to the previous results. The median period of follow-up was calculated for the entire study cohort according to the reverse Kaplan–Meier method. Time to event or final status check was taken from the date of SDCT examination. Receiver operating characteristic curve (ROC curve) and area under the curve (AUC) were used to evaluate the prediction model. ROC curve and Youden index determined the optimal cut-off value of CT-ECV.

Kaplan–Meier curve was used to display the distribution of events based on CT-ECV categories. The log-rank test was applied to compare survival curves between different CT-ECV categories. This analysis enables us to evaluate the relationship between MACE and CT-ECV by determining whether there are significant differences in event-free survival among the categorized ECV groups.

## Results

### Population baseline characteristics

The validation group (G_v_) enrolled 45 patients (45 ± 14 years old, 15 females) (Supplementary Figure 2). The experimental group (G_e_) enrolled 117 subjects, including 82 patients with NIHF and 35 controls, the number of patients in different subgroups within the study population is provided in Supplementary Figure 3. Table 2 summarizes the baseline clinical characteristics of the enrolled subjects, which show that there were no significant differences in age, sex, body mass index, heart rate, and hematocrit level between patients with NIHF and controls.

**Table 2:** Participant characteristics

	All subjects (n = 117)	Patients with NIHF (n = 82)	Controls (n = 35)	*P* value
Age (years)	56.7 ± 11.7	57.6 ± 13.4	54.4 ± 5.91	0.07
Male sex (n,%)	59 (50.4%)	41 (50.0%)	18 (51.4%)	0.371
Heart rate (beats/min)	77.0 ± 14.4	77.7 ± 15.7	74.9 ± 9.50	0.373
Body Mass Index (kg/m^2^)	23.6 ± 3.70	23.9 ± 4.00	22.6 ± 2.70	0.052
Hematocrit level (%)	43.3 ± 6.2	43.5 ± 6.4	42.8 ± 5.7	0.651
BNP (ng/L)	–	532.2 ± 851.6	–	–
NT-proBNP (ng/L)	–	694.3 ± 482.2	–	–
Medical history				
Hypertension (n,%)	34 (29.1%)	27 (32.9%)	7 (20.0%)	0.187
Diabetes mellitus (n,%)	5 (4.27%)	4 (4.88%)	1 (2.86%)	1.000
Smoking (n,%)	18 (15.4%)	15 (18.3%)	3 (8.57%)	0.145
Alcohol (n,%)	20 (17.1%)	19 (23.2%)	1 (2.86%)	< 0.05
Echocardiographic measurements				
LVEF (%)	55.6 ± 17.6	48.3 ± 17.0	69.2 ± 7.39	< 0.05
LVEDV (ml)	153.1 ± 68.1	174.3 ± 70.2	105.3 ± 27.7	< 0.05
LVESV (ml)	74.6 ± 58.8	94.5 ± 61.0	31.3 ± 12.4	< 0.05
LVEDD (mm)	51.6 ± 11.5	56.0 ± 11.1	42.0 ± 4.31	< 0.05
LVESD (mm)	37.8 ± 13.4	43.5 ± 13.5	27.7 ± 4.19	< 0.05
LAV index (mm)	36.7 ± 9.66	41.2 ± 8.02	27.0 ± 3.87	< 0.05
E/A ratio	1.27 ± 1.29	1.16 ± 0.73	1.47 ± 1.92	0.261
E/e' ratio	16.4 ± 7.02	20.3 ± 4.31	7.26 ± 0.61	< 0.05
E' (cm/s)	5.81 ± 2.12	4.56 ± 0.81	8.74 ± 1.12	< 0.05
TR peak velocity (m/s)	2.73 ± 0.40	2.98 ± 0.45	2.22 ± 0.05	< 0.05
PASP (mmHg)	34.8 ± 14.7	38.9 ± 15.1	23.7 ± 3.79	< 0.05
AV peak gradient (mmHg)	9.38 ± 3.76	10.9 ± 4.93	5.96 ± 0.18	< 0.05

Data are expressed as mean ± standard deviation or number of patients with percentages in parentheses, as appropriate

NIHF, non-ischemic heart failure; LV, left ventricle; EF, ejection fraction; EDV, end-diastolic volume; ESV, end-systolic volume; EDD, end-diastolic dimension; ESD, end-systolic dimension; LAV, left atrium volume; TR peak velocity, tricuspid regurgitation peak velocity; PASP, pulmonary artery systolic pressure; AV, aortic valve

*P* < 0.05 indicates a significant difference between controls and patients with non-ischemic heart failure

### Coronary artery degree of stenosis and coronary artery calcium score calculation

The degree of coronary artery stenosis in 82 patients with non-ischemic heart failure was evaluated as follows: Among 82 patients with NIHF, 44 patients showed no visible stenosis (44/82, 54%), 21 patients had minimal stenosis (1–24%) (21/82, 26%), and 17 patients had mild stenosis (25–49%) (17/82, 20%). Coronary artery calcium scores were calculated from pre-contrast images. 63 patients had no calcified plaques (63/82, 76.8%), 18 patients exhibited mild coronary artery calcification (1–100) (18/82, 22.0%), and 1 patient had moderate coronary artery calcification (101 – 300) (1/82, 1.2%).

### Radiation dose

The mean radiation dose of our cardiac SDCT protocol and LIE images was 8.96 ± 1.78 mSv and 2.22 ± 0.53 mSv, respectively (Supplementary Table 2).

### CT-ECV vs. CMR-ECV in the validation group

In the validation group (Gv), a total of 720 segments of the LV myocardium were analyzed, and 635 (88.19%) of these segments met the analytical requirements.

Segment-based analysis showed that there was no significant difference between CT-ECV and CMR-ECV (all *P* > 0.05). The CT-ECV and CMR-ECV per-segment values were well correlated (all *P* < 0.05) (Supplementary Table 3).

Layer-based analysis showed that the differences between CT-ECV and CMR-ECV were not statistically significant (all *P* > 0.05). In addition, CT-ECV was correlated with CMR-ECV (all *P* < 0.05). Bland–Altman analysis showed that CT-ECV matched well with CMR-ECV, with an insignificant bias (Supplementary Table 4).

Global-based analysis showed that there was no significant difference between CT-ECV and CMR-ECV (33.10 ± 4.22% vs. 32.80 ± 4.90%; *P* = 0.293) and that CT-ECV was well correlated with CMR-ECV (r = 0.926; *P* < 0.05), with a small bias (− 0.30%; 95% CI − 3.97% to 3.38%).

### Reproducibility of CT-ECV

The inter- and intra-observer agreement levels for CT-ECV were 0.955 (95% CI 0.919–0.976) and 0.949 (95% CI 0.907–0.972), with wide confidence limits and small biases for inter-observer (bias = − 0.12%; 95% CI − 2.96% to 2.72%) and intra-observer agreement (bias = − 0.16%; 95% CI − 2.19% to 1.87%), respectively.

### CT-ECV of patients with NIHF and controls

Segment-based analysis showed that CT-ECV in patients with NIHF was higher than in controls (Supplementary Table 5).

Layer-based analysis showed that CT-ECV in patients with NIHF was significantly higher than in controls (all *P* < 0.001) (Supplementary Table 6).

**Fig. 2:**
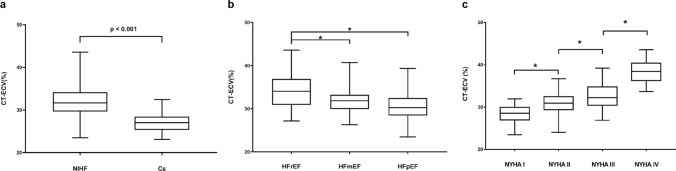
CT-ECV values of patients with NIHF and controls. **a** CT-ECV in patients with non-ischemic heart failure (NIHF) was significantly higher than that found in controls (32.08 ± 4.06% vs. 26.95 ± 2.25%; *P* < 0.001). **b** CT-ECV in HFrEF patients was statistically higher than that of HFmEF and HFpEF patients (HFrEF: 34.04 ± 4.01%, HFmEF: 31.80 ± 3.18%, HFpEF: 30.72 ± 3.79%; *: *P* < 0.05). **c** CT-ECV differences among patients with NIHF with varied New York Heart Association (NYHA) Classes (NYHA I: 28.33 ± 2.28%, NYHA II: 30.60 ± 2.65%, NYHA III: 32.87 ± 3.24%, NYHA IV: 38.30 ± 2.76%; *: *P* < 0.05). Cs, controls

Global-based analysis showed that CT-ECV in patients with NIHF was significantly higher than in controls (32.08 ± 4.06% vs. 26.95 ± 2.25%; *P* < 0.001) (Fig. 2a).

**Fig. 3:**
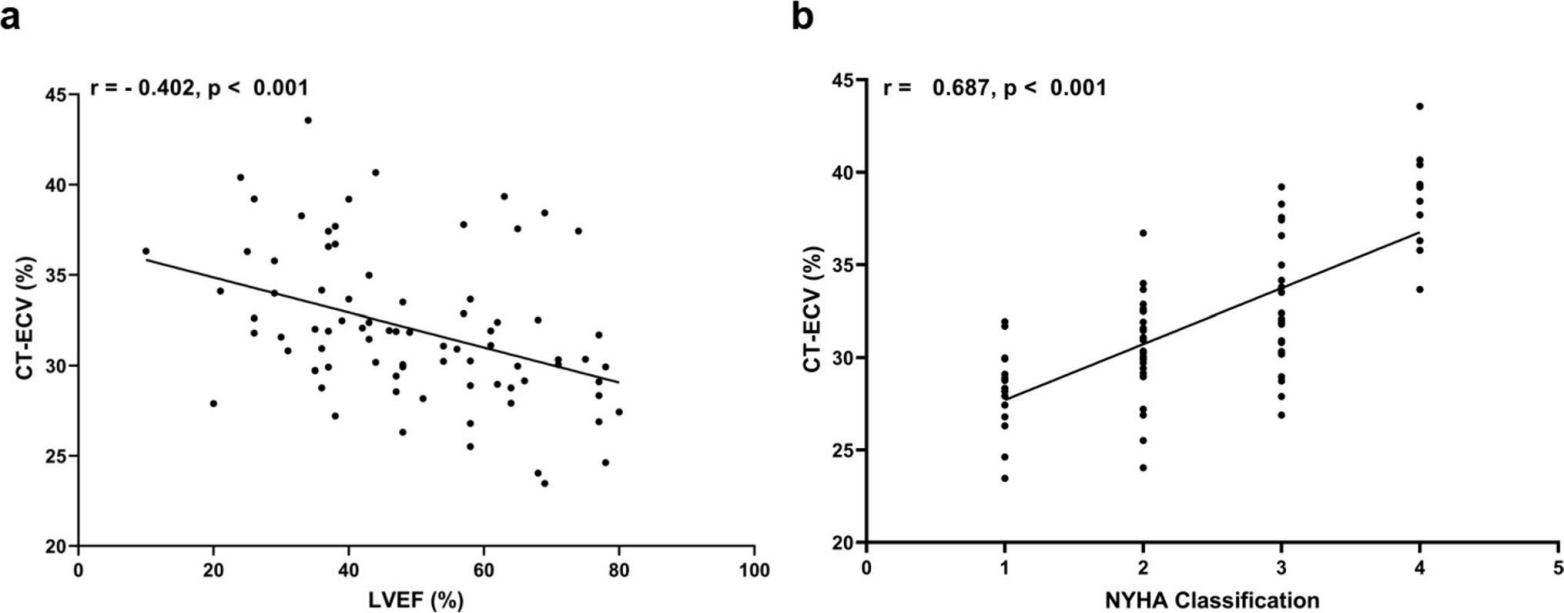
Correlations between CT-ECV, LVEF and NYHA classification. **a** CT-ECV had a negative correlation with left ventricular ejection fraction (r = – 0.402; *P* < 0.001). **b** and a positive correlation with NYHA classification (r = 0.687; *P* < 0.001)

Subgroup-based analysis of patients with NIHF showed that CT-ECV in HFrEF patients was statistically higher than that of HFmEF and HFpEF patients (HFrEF: 34.04 ± 4.01%, HFmEF: 31.80 ± 3.18%, HFpEF: 30.72 ± 3.79%) (Fig. 2b). It also showed CT-ECV differences between patients with NIHF with varied NYHA Classes (NYHA I: 28.33 ± 2.28%, NYHA II: 30.60 ± 2.65%, NYHA III: 32.87 ± 3.24%, and NYHA IV: 38.30 ± 2.76%; all *P* < 0.05) (Fig. 2c). CT-ECV was negatively correlated with LVEF (r = − 0.402; *P* < 0.001) and positively correlated with NYHA Classification (r = 0.687; *P* < 0.001) (Fig. 3). Supplementary Table 7 and Table 8 summarizes the baseline clinical characteristics of subgroups.

### Clinical outcomes

**Fig. 4:**
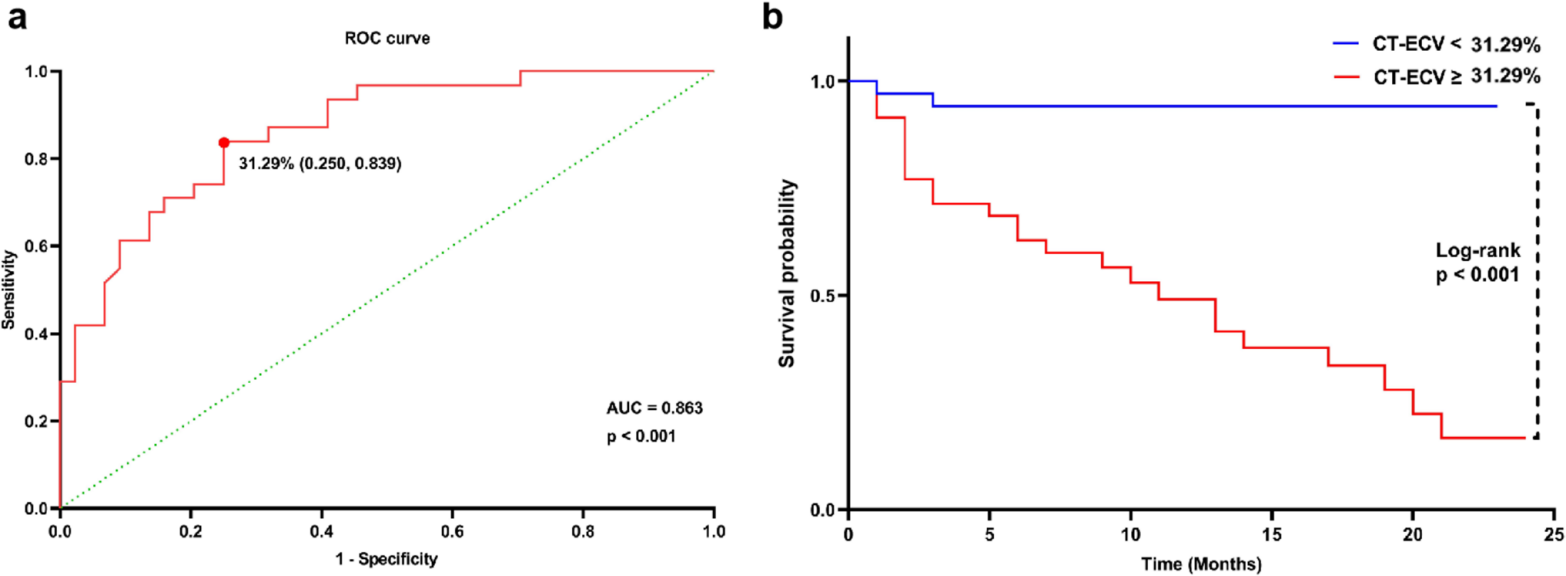
Correlation of CT-ECV and MACE. **a** Receiver operating characteristic (ROC) curves of CT-ECV against MACE. Cut-off value = 31.29%. **b** Kaplan–Meier curve of MACE probability based on CT-ECV level. AUC, area under the curve

Clinical outcome data were collected from 82 patients with NIHF after a median follow up of 10 months (interquartile range: 5 to 13). Final status check was performed during March 2023, and 7 patients lost follow-up. Among the 75 patients with NIHF, 31 patients experienced MACE (31/75, 41.3%), including 28 patients with NIHF who were hospitalized for heart failure (28/75, 37.3%), 1 patient with NIHF experienced cardiovascular mortality (28/75, 1.3%), and 2 patients with NIHF experienced all-cause mortality (2/75, 2.7%). The ROC curves demonstrated CT-ECV ≥ 31.29% to be the optimal cut-off point for MACE with 83.9% sensitivity, 75% specificity and the area under the ROC curve = 0.863 (95% CI 0.782 to 0.944) (Fig. 4a). Kaplan–Meier survival curves and Log-rank test demonstrated that NIHF patients with CT-ECV ≥ 31.29% had higher probability of MACE than NIHF patients with CT-ECV < 31.29% (Fig. 4b).

## Discussion

In the current study, we calculated LIE-derived ECV on iodine density images using SDCT, and we demonstrated the following: (1) that CT-ECV had good reproducibility and accuracy, potentially serving as a promising and practical alternative to CMR-ECV; and (2) that CT-ECV had the potential to assist with risk stratification among patients with NIHF.

Currently, ECV derived from CMR T1 mapping, as an established modality, is based on the T1 relaxation time of the LV myocardium and the blood pool before and after gadolinium injection. Gadolinium is an extracellular agent that is distributed in the extracellular space. Normally, within seconds or minutes of intravenous administration, contrast materials are “washed into” the extracellular space without entering into the cell and then “washed out” from the extracellular space several minutes after injection. However, in a variety of cardiomyopathies, diffuse myocardial fibrosis develops into the expansion of the extracellular matrix, which then causes contrast materials to accumulate more in the extracellular space. The extracellular contrasts are then “washed out” more slowly [8], eventually generating theoretically measurable signal changes that can be assessed via the application of magnetic resonance imaging.

LIE-derived CT-ECV has demonstrated its potential to quantify diffuse myocardial fibrosis in some previous studies based on iodine contrast media, which have kinetics similar to those of gadolinium [9, 25]. Bandula et al. [6] developed and validated an equilibrium contrast-enhanced CT protocol that involved continuous infusion after bolus injection and demonstrated that CT-ECV in patients with aortic stenosis was strongly correlated with the histological quantification of myocardial fibrosis and with CMR-ECV. These previous studies indicated that CT-ECV was comparable to CMR-ECV and correlated with histological collagen volume fraction. However, these prior studies have some limitations, such as cumbersome processes, restricted application, and insufficiently comprehensive analysis. In the current study, we demonstrated that CT-ECV–derived iodine density images using SDCT showed good reproducibility and correlated well enough with CMR-ECV, which is in line with the results of previous studies [6, 9]. Moreover, we have managed to remedy the shortcomings of those previous studies. First, for example, we used the head-to-head comparison of CT-ECV and CMR-ECV on a per-segment, per-layer (basal, mid, and apical) basis to accurately and meticulously evaluate the feasibility of CT-ECV. Second, we measured CT-ECV to obviate ROIs drawn on pre-contrast images, which avoided the sophisticated registration between pre- and post-contrast images and thus reduced misregistration errors. Third, the application of CT-ECV is simplified and routine without additional prospective decisions.

As compared with previous studies, the present study has further explored the value of CT-ECV for risk stratification among patients with NIHF. It was found that CT-ECV among patients with NIHF was significantly higher than that among controls. This finding indicates that increased CT-ECV implies the presence of diffuse fibrosis and that it is feasible to use CT-ECV as a diagnostic index in clinical settings to differentiate healthy subjects from patients with disease [21, 26]. In addition, higher CT-ECV values are found in patients with more severe HF, thus demonstrating that the remodeling of the extracellular matrix or myocardial fibrosis is likely associated with a decline in ventricular function across different types of NIHF and that CT-ECV can reflect the course of disease.

Furthermore, we demonstrated that, in patients with NIHF, CT-ECV remains a significant outcome predictor. Thus, in the assessment of patients with NIHF, CT-ECV may play a valuable role in risk stratifying patients with patients with NIHF in clinical setting.

With the advent of advanced CT technologies like dual-energy CT, spectral CT, and even photon-counting CT, the application of coronary CT angiography (CCTA) in cardiovascular diseases has broadened significantly beyond traditional coronary artery assessments. CCTA now allows comprehensive evaluation of cardiac function (e.g., LVEF, myocardial strain), myocardial perfusion and fibrosis [27–29]. CT-based measurements, like global longitudinal strain, are particularly useful in patients with congenital valve disease or coronary stenosis and have been shown to be more sensitive in detecting early systolic dysfunction [27, 28]. Virtual single-energy imaging using spectral detector CT (SDCT) or photon-counting CT dramatically improves the visualization of coronary arteries, offering superior image quality with reduced contrast media, without increasing radiation exposure [30]. Additionally, SDCT enables precise mapping of iodine distribution during first-pass myocardial perfusion, which provides essential data for diagnosing ischemic and non-ischemic cardiomyopathy. By evaluating coronary flow reserve (e.g., FFRCT ≤ 0.75) in conjunction with myocardial iodine perfusion, SDCT delivers critical functional information crucial for coronary artery disease management [31]. Moreover, late iodine enhancement imaging is valuable in detecting myocardial fibrosis in both ischemic and non-ischemic conditions (e.g., hypertrophic cardiomyopathy, aortic stenosis) [26, 32]. Our research demonstrates that LIE-derived CT-ECV is a reliable quantitative tool for assessing diffuse fibrosis in non-ischemic heart failure, with high reproducibility and correlation in predicting MACE. This contributes a crucial piece to completing the puzzle of one-stop cardiac examination via CCTA. Thus, advancements in CT technology offer a more holistic evaluation of cardiac disease, aiding clinicians in making more informed treatment decisions.

During CT-ECV evaluation, the timing of LIE scanning after contrast injection is crucial. The optimal timing for LIE has not been universally determined, but studies have suggested different timings ranging from 5 to 15 min after injection [7, 8, 10]. In our study, we selected 7 min after contrast injection as the optimal timing for LIE due to its superior image quality and efficiency. However, further verification is needed through future animal experiments to determine the optimal timing for LIE using SDCT.

In our study, the validation group (Gv) consisted of 45 patients who underwent both echocardiographic and cardiac magnetic resonance (CMR) assessments of left ventricular ejection fraction (LVEF). The results indicated that the LVEF assessed by echocardiography was significantly higher than that by CMR (48.72 ± 18.45% vs. 38.02 ± 22.44%, *P* < 0.001). These discrepancies may stem from differences in imaging modalities and assessment techniques. Previous studies have demonstrated that echocardiography tends to overestimate LVEF in patients with severe heart failure, especially those with dilated cardiomyopathy (CMR: 46 ± 14% vs. 2D Echo: 56 ± 15%, *P* < 0.001) [33]. While CMR is considered the gold standard for evaluating ventricular volumes and cardiac function [34], current international guidelines predominantly rely on echocardiographic assessments when classifying heart failure patients [15]. Therefore, we classified the validation group in our subsequent studies based on echocardiographic results to align with clinical practice. In view of this, it is necessary for future research to further explore the feasibility of incorporating CMR into routine clinical practice. This would contribute to enhancing the accuracy of heart failure classification, optimizing patient management, and providing a more reliable basis for clinical decision-making.

### Study limitations

First, CT-ECV involves exposure to additional radiation. Nevertheless, the mean radiation dose of our cardiac SDCT protocol is lower than that of previous studies [5, 10]. In addition, since the combination of LIE with CCTA in our study accomplished the evaluation of cardiac structure and function, the coronary arteries, and tissue characteristics in a “one-stop shop” examination, it offers more clinical benefits to patients with known or suspected HF, which may offset some of its drawback. Third, because our CT-ECV results were not validated by the gold standard of histology biopsy, we performed a head-to-head comparison with CMR-ECV, a well-recognized gold standard for the noninvasive evaluation of diffuse myocardial fibrosis. This was done to validate the feasibility of CT-ECV. Last, we did not establish and analyze CT-ECV in different disease subgroups, but this also makes CT-ECV applicable to a wider range of diseases. In our future studies, we will further explore CT-ECV in disease surveillance and prognostic assessment for different cardiac diseases.

## Conclusion

In conclusion, this study shows that ECV derived from LIE on iodine density images using SDCT is a promising practical alternative to CMR-ECV for the noninvasive quantification of diffuse myocardial fibrosis and has the potential to assist with risk stratification among patients with NIHF.

## Supplementary Information

Below is the link to the electronic supplementary material.

Supplementary file1 (DOCX 840 KB)

## Abbreviations

ECV: Extracellular volume fraction
LIE: Late iodine enhancement
LV: Left ventricular
LVEF: Left ventricular ejection fraction
NIHF: Non-ischemic heart failure
NYHA: New York Heart Association
ROIs: Regions of interest
SDCT: Spectral detector computed tomography
CMR: Cardiac magnetic resonance
SBI: Spectral base image

## References

[1] Mewton N, Liu CY, Croisille P, Bluemke D, Lima JA (2011) Assessment of myocardial fibrosis with cardiovascular magnetic resonance. J Am Coll Cardiol 57(8):891–903 21329834 10.1016/j.jacc.2010.11.013 PMC3081658

[2] Moon JC, Messroghli DR, Kellman P, Piechnik SK, Robson MD, Ugander M, Gatehouse PD, Arai AE, Friedrich MG, Neubauer S (2013) Myocardial T1 mapping and extracellular volume quantification: a Society for Cardiovascular Magnetic Resonance (SCMR) and CMR Working Group of the European Society of Cardiology consensus statement. J Cardiovasc Magn Reson 15(1):92. 10.1186/1532-429X-15-92 24124732 10.1186/1532-429X-15-92 PMC3854458

[3] Yang EY, Ghosn MG, Khan MA, Gramze NL, Brunner G, Nabi F, Nambi V, Nagueh SF, Nguyen DT, Graviss EA (2019) Myocardial extracellular volume fraction adds prognostic information beyond myocardial replacement fibrosis. Circ Cardiovasc Imaging 12(12):e009535. 10.1161/CIRCIMAGING.119.009535 31838882 10.1161/CIRCIMAGING.119.009535 PMC7529265

[4] Meduri A, Perazzolo A, Marano R, Muciaccia M, Lauriero F, Rovere G, Giarletta L, Moliterno E, Natale L (2024) Cardiac MRI in heart failure with preserved ejection fraction. Radiol Med 129(10):1468–1484 39158816 10.1007/s11547-024-01874-z

[5] Oda S, Emoto T, Nakaura T, Kidoh M, Utsunomiya D, Funama Y, Nagayama Y, Takashio S, Ueda M, Yamashita T (2019) Myocardial late iodine enhancement and extracellular volume quantification with dual-layer spectral detector dual-energy cardiac CT. Radiol Cardiothorac Imaging 1(1):e180003. 10.1148/ryct.2019180003 33778497 10.1148/ryct.2019180003 PMC7977749

[6] Bandula S, White SK, Flett AS, Lawrence D, Pugliese F, Ashworth MT, Punwani S, Taylor SA, Moon JC (2013) Measurement of myocardial extracellular volume fraction by using equilibrium contrast-enhanced CT: validation against histologic findings. Radiol 269(2):396–403. 10.1148/radiol.13130130 10.1148/radiology.13130130 23878282

[7] Kurita Y, Kitagawa K, Kurobe Y, Nakamori S, Nakajima H, Dohi K, Ito M, Sakuma H (2016) Estimation of myocardial extracellular volume fraction with cardiac CT in subjects without clinical coronary artery disease: a feasibility study. J Cardiovasc Comput Tomogr 10(3):237–241. 10.1016/j.jcct.2016.02.001 26968674 10.1016/j.jcct.2016.02.001

[8] Scully PR, Bastarrika G, Moon JC, Treibel TA (2018) Myocardial extracellular volume quantification by cardiovascular magnetic resonance and computed tomography. Curr Cardiol Rep 20:1–11 29511861 10.1007/s11886-018-0961-3 PMC5840231

[9] Zhou Z, Gao Y, Wang H, Wang W, Zhang H, Wang S, Sun Z, Xu L (2021) Myocardial extracellular volume fraction analysis in doxorubicin-induced beagle models: comparison of dual-energy CT with equilibrium contrast-enhanced single-energy CT. Cardiovasc Diagn Ther 11(1):102 33708482 10.21037/cdt-20-798 PMC7944207

[10] Abadia AF, van Assen M, Martin SS, Vingiani V, Griffith LP, Giovagnoli DA, Bauer MJ, Schoepf UJ (2020) Myocardial extracellular volume fraction to differentiate healthy from cardiomyopathic myocardium using dual-source dual-energy CT. J Cardiovasc Comput Tomogr 14(2):162–167. 10.1016/j.jcct.2019.09.008 31615736 10.1016/j.jcct.2019.09.008

[11] Rajiah P, Abbara S, Halliburton SS (2017) Spectral detector CT for cardiovascular applications. Diagn Interv Radiol 23(3):187. 10.5152/dir.2016.16255 28302592 10.5152/dir.2016.16255 PMC5410998

[12] Majeed NF, Braschi Amirfarzan M, Wald C, Wortman JR (2021) Spectral detector CT applications in advanced liver imaging. Br J Radiol 94(1123):20201290. 10.1259/bjr.20201290 34048285 10.1259/bjr.20201290 PMC8248211

[13] Narula J, Chandrashekhar Y, Ahmadi A, Abbara S, Berman DS, Blankstein R, Leipsic J, Newby D, Nicol ED, Nieman K (2020) SCCT 2021 expert consensus document on coronary computed tomographic angiography: a report of the society of cardiovascular computed tomography. J Cardiovasc Comput Tomogr 15(3):192. 10.1016/j.jcct.2020.11.001 33303384 10.1016/j.jcct.2020.11.001 PMC8713482

[14] Cury RC, Leipsic J, Abbara S, Achenbach S, Berman D, Bittencourt M, Budoff M, Chinnaiyan K, Choi AD, Ghoshhajra B (2022) CAD-RADS™ 2.0–2022 coronary artery disease-reporting and data system: an expert consensus document of the society of cardiovascular computed tomography (SCCT), the American college of cardiology (ACC), the American college of radiology (ACR), and the North America society of cardiovascular imaging (NASCI). Cardiovasc Imaging 15(11):1974–2001. 10.1016/j.jcmg.2022.07.002 10.1016/j.jcmg.2022.07.002 36115815

[15] McDonagh TA, Metra M, Adamo M, Gardner RS, Baumbach A, Böhm M, Burri H, Butler J, Čelutkienė J, Chioncel O (2021) 2021 ESC Guidelines for the diagnosis and treatment of acute and chronic heart failure: Developed by the Task Force for the diagnosis and treatment of acute and chronic heart failure of the European Society of Cardiology (ESC) With the special contribution of the Heart Failure Association (HFA) of the ESC. Eur Heart J 42(36):3599–3726. 10.1093/eurheartj/ehab368 34447992 10.1093/eurheartj/ehab368

[16] Bozkurt B, Colvin M, Cook J, Cooper LT, Deswal A, Fonarow GC, Francis GS, Lenihan D, Lewis EF, McNamara DM (2016) Current diagnostic and treatment strategies for specific dilated cardiomyopathies: a scientific statement from the American Heart Association. Circulation 134(23):e579–e646. 10.1161/CIR.0000000000000455 27832612 10.1161/CIR.0000000000000455

[17] Georgiopoulou VV, Kalogeropoulos AP, Raggi P, Butler J (2010) Prevention, diagnosis, and treatment of hypertensive heart disease. Cardiol Clin 28(4):675–691 20937450 10.1016/j.ccl.2010.07.005

[18] Hayat SA, Patel B, Khattar RS, Malik RA (2004) Diabetic cardiomyopathy: mechanisms, diagnosis and treatment. Clin Sci 107(6):539–557. 10.1042/CS20040057 10.1042/CS20040057 15341511

[19] Nishimura RA, Otto CM, Bonow RO, Carabello BA, Erwin JP III, Fleisher LA, Jneid H, Mack MJ, McLeod CJ, O’Gara PT (2017) 2017 AHA/ACC focused update of the 2014 AHA/ACC guideline for the management of patients with valvular heart disease: a report of the American College of Cardiology/American Heart Association Task Force on Clinical Practice Guidelines. Circulation 135(25):e1159–e1195. 10.1161/CIR.000000000000050 28298458 10.1161/CIR.0000000000000503

[20] Richardson P (1996) Report of the 1995 world health organization/international society and federation of cardiology task Force on the definition and classification of cardiomyopathies. Circulation 93:841–842 8598070 10.1161/01.cir.93.5.841

[21] Fischer K, Obrist SJ, Erne SA, Stark AW, Marggraf M, Kaneko K, Guensch DP, Huber AT, Greulich S, Aghayev A (2020) Feature tracking myocardial strain incrementally improves prognostication in myocarditis beyond traditional CMR imaging features. Cardiovasc Imaging 13(9):1891–1901. 10.1016/j.jcmg.2020.04.025 10.1016/j.jcmg.2020.04.025 32682718

[22] Halliburton SS, Abbara S, Chen MY, Gentry R, Mahesh M, Raff GL, Shaw LJ, Hausleiter J (2011) SCCT guidelines on radiation dose and dose-optimization strategies in cardiovascular CT. J Cardiovasc Comput Tomogr 5(4):198–224. 10.1016/j.jcct.2011.06.001 21723512 10.1016/j.jcct.2011.06.001 PMC3391026

[23] Li Z, Han D, Qi T, Deng J, Li L, Gao C, Gao W, Chen H, Zhang L, Chen W (2023) Hemoglobin A1c in type 2 diabetes mellitus patients with preserved ejection fraction is an independent predictor of left ventricular myocardial deformation and tissue abnormalities. BMC Cardiovasc Disord 23(1):49 36698087 10.1186/s12872-023-03082-5 PMC9878773

[24] Lang RM, Badano LP, Mor-Avi V, Afilalo J, Armstrong A, Ernande L, Flachskampf FA, Foster E, Goldstein SA, Kuznetsova T (2015) Recommendations for cardiac chamber quantification by echocardiography in adults: an update from the American Society of Echocardiography and the European Association of Cardiovascular Imaging. Eur Heart J Cardiovasc Imaging 16(3):233–271. 10.1093/ehjci/jev014 25712077 10.1093/ehjci/jev014

[25] Hong YJ, Kim TK, Hong D, Park CH, Yoo SJ, Wickum ME, Hur J, Lee H-J, Kim YJ, Suh YJ (2016) Myocardial characterization using dual-energy CT in doxorubicin-induced DCM: comparison with CMR T1-mapping and histology in a rabbit model. J Am Coll Cardiol Img 9(7):836–845. 10.1016/j.jcmg.2015.12.018 10.1016/j.jcmg.2015.12.018 27236517

[26] Lee H-J, Im DJ, Youn J-C, Chang S, Suh YJ, Hong YJ, Kim YJ, Hur J, Choi BW (2016) Myocardial extracellular volume fraction with dual-energy equilibrium contrast-enhanced cardiac CT in nonischemic cardiomyopathy: a prospective comparison with cardiac MR imaging. Radiology 280(1):49–57. 10.1148/radiol.2016151289 27322972 10.1148/radiol.2016151289

[27] Vach M, Vogelhuber J, Weber M, Sprinkart AM, Pieper CC, Block W, Kuetting D, Attenberger UI, Luetkens JA (2021) Feasibility of CT-derived myocardial strain measurement in patients with advanced cardiac valve disease. Sci Rep 11(1):8793 33888835 10.1038/s41598-021-88294-5 PMC8062484

[28] Han X, Cao Y, Ju Z, Liu J, Li N, Li Y, Liu T, Shi H, Gu J (2020) Assessment of regional left ventricular myocardial strain in patients with left anterior descending coronary stenosis using computed tomography feature tracking. BMC Cardiovasc Disord 20:1–11 32770941 10.1186/s12872-020-01644-5 PMC7414558

[29] Nieman K, Balla S (2020) Dynamic CT myocardial perfusion imaging. J Cardiovasc Comput Tomogr 14(4):303–306. 10.1016/j.ejrad.2016.07.017 31540820 10.1016/j.jcct.2019.09.003 PMC7064397

[30] D’Angelo T, Lanzafame LR, Micari A, Blandino A, Yel I, Koch V, Gruenewald LD, Vogl TJ, Booz C, Bucolo GM (2023) Improved coronary artery visualization using virtual monoenergetic imaging from dual-layer spectral detector CT angiography. Diagnostics 13(16):2675. 10.3390/diagnostics13162675 37627934 10.3390/diagnostics13162675 PMC10453590

[31] D’Angelo T, Martin S, Micari A, Booz C, Steyer A, Blandino A, Lanzafame LR, Koch V, Ascenti G, Mazziotti S (2022) Coronary angiography using spectral detector dual-energy CT: Is it the time to assess myocardial first-pass perfusion? Eur Radiol Exp 6(1):60 36480065 10.1186/s41747-022-00313-w PMC9732170

[32] Dubourg B, Dacher J-N, Durand E, Caudron J, Bauer F, Bubenheim M, Eltchaninoff H, Serfaty J-M (2021) Single-source dual energy CT to assess myocardial extracellular volume fraction in aortic stenosis before transcatheter aortic valve implantation (TAVI). Diagn Interv Imaging 102(9):561–570. 10.1016/j.diii.2021.03.003 33903056 10.1016/j.diii.2021.03.003

[33] Hoffmann R, Barletta G, Von Bardeleben S, Vanoverschelde JL, Kasprzak J, Greis C, Becher H (2014) Analysis of left ventricular volumes and function: a multicenter comparison of cardiac magnetic resonance imaging, cine ventriculography, and unenhanced and contrast-enhanced two-dimensional and three-dimensional echocardiography. J Am Soc Echocardiogr 27(3):292–301. 10.1016/j.echo.2013.12.005 24440110 10.1016/j.echo.2013.12.005

[34] Greupner J, Zimmermann E, Grohmann A, Dübel H-P, Althoff T, Borges AC, Rutsch W, Schlattmann P, Hamm B, Dewey M (2012) Head-to-head comparison of left ventricular function assessment with 64-row computed tomography, biplane left cineventriculography, and both 2-and 3-dimensional transthoracic echocardiography: comparison with magnetic resonance imaging as the reference standard. JACC 59(21):1897–1907. 10.1016/j.jacc.2012.01.046 22595410 10.1016/j.jacc.2012.01.046

