# Supplementary material for: Myocardial extracellular volume fraction with spectral detector computed tomography for risk stratification in non-ischemic heart failure
Source: Radiol Med. 2025 Apr 30;130(7):1092–104. doi: 10.1007/s11547-025-02002-1 (PMC12263805; doi:10.1007/s11547-025-02002-1)
Supplement: Supplementary file 1 — Supplementary file1 (DOCX 840 KB) [file 11547_2025_2002_MOESM1_ESM.docx]

**Supplementary Table 1: The acquisition parameters of the cardiac magnetic resonance protocol**

|  | **Cine** | **T1 mapping** | **LGE** |
| --- | --- | --- | --- |
| Sequence | B-SSFP | MOLLI | PSIR |
| TR/TE (ms) | 42/1.4 | 311/1.3 | 636/3.1 |
| Flip angle (°) | 60 | 35 | 25 |
| Display field of view (mm^2^) | 330×330 | 330×330 | 330×330 |
| Voxel | 170×172 | 200 × 160 | 200×152 |
| Slice thickness (mm) | 8 | 8 | 8 |
| Slice gap (mm) | 2 | 2 | 2 |

**Supplementary Table 2: Dose-length product and estimated effective radiation dose of the study**

| **Study acquisition** | **Dose length product (mGy*cm)** | **Estimated effective radiation dose (mSv)** |
| --- | --- | --- |
| Pre-contrast CT | 55.4 ± 9.7 (43.9-86.0) | 0.77 ± 0.14 (0.61-1.20) |
| CCTA | 425.7 ± 105.7 (261.0-688.5) | 5.96± 1.48 (3.65-9.64) |
| LIE | 158.8± 38.2 (120.0-265.9) | 2.22 ± 0.53 (1.7-3.7) |
| Entire study protocol | 639.9 ± 127.2 (427.0-952.5) | 8.96 ± 1.78 (6.0-13.34) |

Note: Data are expressed as mean ± standard deviation, with ranges given in parentheses. The effective radiation dose was calculated using the dose-length product multiplied by a chest conversion coefficient (k = 0.014 mSv/mGy/cm) in accordance with the computed tomography criteria of the European Working Group Guidelines [21].

**Supplementary Table 3: Segment-based results of correlation, comparison, and Bland-Altman analysis between CT-ECV (%) and CMR-ECV (%)**

| **AHA** | **CT-ECV**  **（%）** | **CMR-ECV**  **（%）** | **Correlation** | **P ^a^ value** | **P^b^ value** | **Bias** | **95%CI** |
| --- | --- | --- | --- | --- | --- | --- | --- |
| 1 | 32.65 ± 5.01 | 32.21 ± 6.51 | 0.768 | <0.001 | 0.498 | 0.44 | (-7.723 to 8.613) |
| 2 | 33.09 ± 5.31 | 32.56 ± 5.32 | 0.559 | <0.001 | 0.509 | 0.53 | (-9.255 to 10.32) |
| 3 | 32.75 ± 4.78 | 30.83 ± 6.32 | 0.463 | 0.002 | 0.053 | 1.91 | (-9.645 to 13.47) |
| 4 | 33.44 ± 6.07 | 32.56 ± 5.32 | 0.729 | <0.001 | 0.199 | 0.89 | (-7.433 to 9.212) |
| 5 | 34.39 ± 5.06 | 35.59 ± 6.43 | 0.720 | <0.05 | 0.103 | -1.20 | (-9.984 to 7.585) |
| 6 | 34.49 ± 5.24 | 36.34 ± 7.09 | 0.495 | 0.001 | 0.075 | -1.85 | (-14.40 to 10.69) |
| 7 | 32.10 ± 5.39 | 33.17 ± 6.49 | 0.733 | <0.001 | 0.136 | -1.07 | (-9.807 to 7.664) |
| 8 | 33.58 ± 4.68 | 32.56 ± 7.39 | 0.769 | <0.001 | 0.179 | 1.01 | (-8.435 to 10.47) |
| 9 | 34.06 ± 5.10 | 33.03 ± 5.57 | 0.768 | <0.001 | 0.086 | 1.02 | (-6.162 to 8.204) |
| 10 | 32.45 ± 5.61 | 32.73 ± 6.17 | 0.738 | <0.001 | 0.691 | -0.28 | (-8.693 to 8.128) |
| 11 | 33.68 ± 5.24 | 33.98 ± 5.37 | 0.724 | <0.001 | 0.632 | -0.30 | (-8.025 to 7.430) |
| 12 | 33.39 ± 5.51 | 34.53 ± 6.64 | 0.674 | <0.001 | 0.163 | -1.13 | (-10.95 to 8.692) |
| 13 | 32.12 ± 4.75 | 33.19 ± 8.81 | 0.796 | <0.001 | 0.264 | 2.67 | (-7.739 to 13.07) |
| 14 | 33.14 ± 4.73 | 31.78 ± 6.09 | 0.509 | 0.008 | 0.158 | 1.36 | (-9.407 to 12.13) |
| 15 | 31.99 ± 5.86 | 32.40 ± 5.19 | 0.675 | <0.001 | 0.604 | -0.41 | (-9.212 to 8.393) |
| 16 | 33.68 ± 5.66 | 35.51 ± 12.1 | 0.628 | <0.001 | 0.278 | -1.82 | (-20.71 to 17.06) |

ECV, extracellular volume fraction; CMR, cardiac magnetic resonance imaging; AHA, American Heart Association's 16 segmentation; CI, confidence interval.

P ^a^ value for the correlation; P ^b^ value for the comparison.

**Supplementary Table 4: Layer-based results of correlation, comparison, and Bland-Altman analysis between CT-ECV (%) and CMR-ECV (%)**

| **Layer** | **CMR-ECV**  **（%）** | **CT-ECV**  **（%）** | **Correlation** | **P ^a^ value** | **P^b^ value** | **Bias** | **95%CI** |
| --- | --- | --- | --- | --- | --- | --- | --- |
| Basal | 32.53 ± 5.47 | 33.27 ± 4.37 | 0.887 | <0.001 | 0.063 | - 0.74 | (- 5.78 to 4.30) |
| Mid | 32.46 ± 4.82 | 33.07 ± 4.32 | 0.905 | <0.001 | 0.051 | - 0.60 | (- 4.64 to 3.41) |
| Apical | 33.04 ± 5.35 | 32.54 ± 5.08 | 0.863 | <0.001 | 0.280 | 0.49 | (- 4.88 to 5.86) |

ECV, extracellular volume fraction; CMR, cardiac magnetic resonance imaging; CI, confidence interval.

P ^a^ value for the correlation; P ^b^ value for the comparison.

**Supplementary Table 5: Comparison of CT-ECV values based on left ventricular myocardium segmentation**

| **AHA** | **Patients with NIHF** | **Controls** | **P value** |
| --- | --- | --- | --- |
| 1 | 30.44 ± 5.66% | 25.64 ± 2.36% | <0.001 |
| 2 | 31.56 ± 4.43% | 27.79 ± 2.88% | <0.001 |
| 3 | 32.07 ± 5.72% | 28.22 ± 3.72% | <0.001 |
| 4 | 32.18 ± 7.68% | 26.18 ± 3.00% | <0.001 |
| 5 | 32.64 ± 6.49% | 27.97 ± 3.36% | <0.001 |
| 6 | 32.13 ± 7.28% | 27.00 ± 3.16% | <0.001 |
| 7 | 29.09 ± 7.25% | 25.53 ± 2.53% | <0.001 |
| 8 | 29.99 ± 7.64% | 27.09 ± 3.08% | <0.001 |
| 9 | 31.37 ± 7.83% | 27.80 ± 3.38% | <0.001 |
| 10 | 30.06 ± 9.32% | 26.32 ± 3.11% | <0.001 |
| 11 | 32.59 ± 6.46% | 27.26 ± 3.19% | <0.001 |
| 12 | 30.73 ± 7.97% | 27.38 ± 3.21% | <0.001 |
| 13 | 28.43 ± 8.08% | 24.22 ± 3.50% | <0.001 |
| 14 | 30.70 ± 7.03% | 26.17 ± 2.87% | <0.001 |
| 15 | 29.07 ± 8.87% | 26.53 ± 3.27% | <0.001 |
| 16 | 30.91 ± 8.62% | 27.46 ± 3.00% | <0.001 |

NIHF, Non-ischemic heart failure; AHA, American Heart Association's 16 segmentation.

**Supplementary Table 6: Comparison of CT-ECV values based on the left ventricular myocardium layer**

| **Layer** | **Patients with NIHF** | **Controls** | **P value** |
| --- | --- | --- | --- |
| Basal | 32.37 ± 4.14% | 27.23 ± 2.41% | < 0.001 |
| Mid | 31.87 ± 4.34% | 26.56 ± 2.73% | < 0.001 |
| Apical | 31.42 ± 4.24% | 26.95 ± 2.25% | < 0.001 |

NIHF, Non-ischemic heart failure.

**Supplementary Table 7: Participant characteristics of subgroups based on LVEF**

|  | **Controls**  **(n = 35)** | **HFrEF**  **(n =30)** | **HFmEF**  **(n = 16)** | **HFpEF**  **(n = 36)** |
| --- | --- | --- | --- | --- |
| Age (years) | 54.4 ±5.91 | 53.4 ±14.8 | 57.1 ±15.2 | 61.4 ±10.1^ab^ |
| Male sex (n,%) | 18 (51.4%) | 21 (70.0%) | 8 (50.0%) | 12 (30.3%)^b^ |
| Heart rate (beats/min) | 74.9 ± 9.50 | 78.8 ± 10.9 | 77.4 ± 14.8 | 72.9 ± 14.1 |
| Body Mass Index ( kg/m²) | 22.6 ± 2.70 | 24.2 ± 4.00 | 24.5 ± 4.79 | 23.5± 3.63 |
| Hematocrit level (%) | 42.8 ± 5.69 | 45.7. ± 5.69 | 44.8 ± 7.48 | 41.1± 5.57^bc^ |
| **Medical history** |  |  |  |  |
| Hypertension (n,%) | 7 (20.0%) | 9 (30.0%) | 7 (43.8%) | 11 (30.6%) |
| Diabetes mellitus (n,%) | 1 (2.86%) | 1 (3.33%) | 0 (0%) | 3 (8.33%) |
| Smoking (n,%) | 3 (8.57%) | 7 (23.3%) | 4 (25.0%) | 4 (11.1%) |
| Alcohol (n,%) | 1 (2.86%) | 9 (30.0%)^a^ | 4 (25.0%)^a^ | 6 (16.7%) |
| LVEF (%) | 69.2 ± 7.39 | 30.8 ± 7.30^a^ | 45.9 ± 6.44^ab^ | 64.5 ± 8.97^abc^ |
| LVEDV (ml) | 105.3 ± 27.7 | 232.3 ± 55.0^a^ | 175.0 ± 67.0^ab^ | 122.6 ± 41.1^abc^ |
| LVESV (ml) | 31.3 ± 12.4 | 148.7 ± 55.5^a^ | 90.6 ± 37.7^ab^ | 47.2 ± 22.7^abc^ |
| LVEDD (mm) | 42.0 ± 4.31 | 65.3 ± 6.97^a^ | 57.4 ± 7.80^ab^ | 46.8 ± 7.78^abc^ |
| LVESD (mm) | 27.7 ± 4.19 | 55.8 ± 8.05^a^ | 45.8 ± 8.41^ab^ | 33.1 ± 8.31^abc^ |
| LAV index (mm) | 27.0 ± 3.87 | 42.1 ± 6.70^a^ | 45.8 ± 8.41^a^ | 40.3 ± 9.93^a^ |
| E/A ratio | 1.47 ± 1.92 | 1.39 ± 0.82 | 1.05 ± 0.80 | 0.99 ± 0.48^b^ |
| E/e' ratio | 7.26 ± 0.61 | 23.9 ± 3.96^a^ | 18.8 ± 1.65^ab^ | 16.9 ± 1.73^abc^ |
| E' (cm/s) | 8.74 ± 1.12 | 4.14 ± 0.54^a^ | 4.56 ± 0.81^ab^ | 5.07 ± 0.83^ab^ |
| TR peak velocity (m/s) | 2.22 ± 0.05 | 3.05 ± 0.40^a^ | 2.55 ± 0.30^ab^ | 2.85 ± 0.30^a^ |
| PASP (mmHg) | 23.7 ± 3.79 | 40.4± 13.5^a^ | 39.1 ± 18.9^a^ | 37.5 ± 14.7^a^ |
| AV peak gradient (mmHg) | 5.96 ± 0.18 | 5.43 ± 1.53 | 8.08 ± 1.99 | 18.7 ± 7.50^abc^ |

Note: Data are expressed as mean ± standard deviation or number of patients with percentages in parentheses, as appropriate. NIHF, Non-ischemic heart failure; LV, left ventricle; EF, ejection fraction; EDV, end-diastolic volume; ESV, end-systolic volume; EDD, end-diastolic dimension; ESD, end-systolic dimension; LAV, left atrium volume; TR peak velocity, tricuspid regurgitation peak velocity; PASP, pulmonary artery systolic pressure; AV, aortic valve.

^a^ P value versus Cs, P < 0.05

^b^ P value versus HFrEF, P < 0.05

^c^ P value versus HFmEF, P < 0.05

P < 0.05 is consider to indicate statistical significance

**Supplementary Table 8: Participant characteristics of subgroups based on NYHA**

|  | **Controls**  **(n = 35)** | **NYHA I**  **(n =16)** | **NYHA II**  **(n = 27)** | **NYHA III**  **(n = 28)** | **NYHA IV**  **(n = 11)** |
| --- | --- | --- | --- | --- | --- |
| Age (years) | 54.4 ±5.91 | 63.9 ±10.8^a^ | 58.7 ±9.03^a^ | 53.9 ±15.7^b^ | 55.2 ±16.9 |
| Male sex (n,%) | 18 (51.4%) | 7 (43.8%) | 13 (48.1%) | 17 (60.7%) | 4 (36.4%) |
| Heart rate (beats/min) | 74.9 ± 9.50 | 79.3 ± 16.1 | 73.0 ± 15.3 | 78.6 ± 14.1 | 81.2 ± 12.5 |
| Bod Mass Index ( kg/m²) | 22.6 ± 2.70 | 24.6 ± 3.52 | 23.1 ± 6.02 | 22.9± 6.35 | 16.5 ± 10.9^b^ |
| Hematocrit level (%) | 42.8 ± 5.7 | 43.0 ± 5.0 | 44.0 ± 7.3 | 44.7 ± 5.8 | 39.5 ± 6.4^d^ |
| **Medical history** |  |  |  |  |  |
| Hypertension (n,%) | 7 (20.0%) | 7 (43.8%) | 11 (40.7%) | 6 (21.4%) | 3 (27.3%) |
| Diabetes mellitus (n,%) | 1 (2.86%) | 1 (6.25%) | 1 (3.70%) | 1 (3.57%) | 1 (9.09%) |
| Smoking (n,%) | 3 (8.57%) | 3 (18.8%) | 3 (11.1%) | 7 (25.0%) | 2 (18.2%) |
| Alcohol (n,%) | 1 (2.86%) | 1 (6.25%) | 5 (18.5%)^a^ | 10 (35.7%)^ab^ | 3 (27.3%) |
| LVEF (%) | 69.2 ± 7.39 | 62.6 ± 12.9 | 51.1 ± 15.0^ab^ | 41.6 ± 15.1^abc^ | 37.8 ± 17.0^abc^ |
| LVEDV (ml) | 105.3 ± 27.7 | 125.3 ± 60.8 | 172.3 ± 65.6^ab^ | 192.4 ± 68.1^ab^ | 199.5± 72.8^ab^ |
| LVESV (ml) | 31.3 ± 12.4 | 49.4 ± 41.8 | 90.2 ± 49.9^ab^ | 104.4 ± 64.5^ab^ | 137.4 ± 66.4^abc^ |
| LVEDD (mm) | 42.0 ± 4.31 | 47.1 ± 8.47^a^ | 55.4 ± 9.74^ab^ | 59.4 ± 10.7^ab^ | 61.5 ± 12.2^ab^ |
| LVESD (mm) | 27.7 ± 4.19 | 29.0 ± 6.72 | 42.9 ± 10.2^ab^ | 49.3 ± 13.6^ab^ | 51.7 ± 11.9^ab^ |
| LAV index (mm) | 27.0 ± 3.87 | 40.3 ± 6.49^a^ | 38.6 ± 7.07^a^ | 42.1 ± 6.85^a^ | 46.8 ± 12.3^ab^ |
| E/A ratio | 1.47 ± 1.92 | 1.01 ± 0.61 | 0.92 ± 0.43 | 1.38 ± 0.79^c^ | 1.67 ± 1.04 |
| E/e' ratio | 7.26 ± 0.61 | 15.4 ± 0.34^a^ | 18.5 ± 0.95^abc^ | 21.4 ± 1.32^abc^ | 29.3 ± 2.22^abcd^ |
| E' (cm/s) | 8.74 ± 1.12 | 5.06 ± 0.85^a^ | 4.85 ± 0.86^a^ | 4.36 ± 0.67^ab^ | 4.07 ± 0.46^abc^ |
| TR peak velocity (m/s) | 2.22 ± 0.05 | 2.32 ± 0.10^a^ | 2.80 ± 0.20^ab^ | 3.10 ± 0.32^ab^ | 3.05 ± 0.40^abc^ |
| PASP (mmHg) | 23.7 ± 3.79 | 36.5± 11.0^a^ | 36.1 ± 16.9^a^ | 38.3 ± 11.2^a^ | 50.4 ± 19.5^ac^ |
| AV peak gradient (mmHg) | 5.96 ± 0.18 | 13.0 ± 5.29 | 12.3 ± 5.24^a^ | 10.4 ± 5.92 | 6.13± 1.35 |

Note: Data are expressed as mean ± standard deviation or number of patients with percentages in parentheses, as appropriate. NIHF, Non-ischemic heart failure; LV, left ventricle; EF, ejection fraction; EDV, end-diastolic volume; ESV, end-systolic volume; EDD, end-diastolic dimension; ESD, end-systolic dimension; LAV, left atrium volume; TR peak velocity, tricuspid regurgitation peak velocity; PASP, pulmonary artery systolic pressure; AV, Aotic Valve.

^a^ P value versus Cs, P < 0.05

^b^ P value versus NYHA I, P < 0.05

^c^ P value versus NYHA II, P < .05

^d^ P value versus NYHA III, P < .05

P < 0.05 is consider to indicate statistical significance


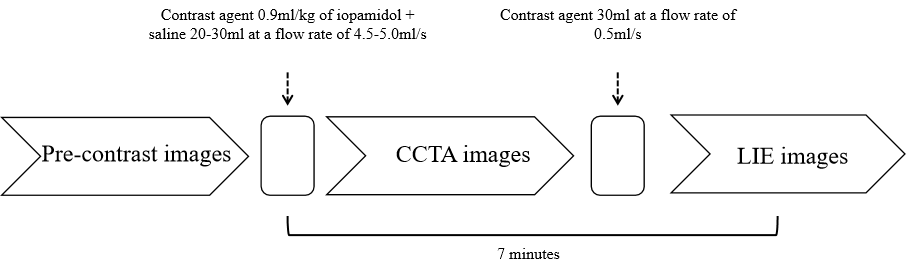


**Supplementary Figure 1:** **Cardiac SDCT scanning protocol**

Pre-contrast images were acquired first, and then coronary computed tomography angiography images were acquired by contrast injection. Finally, late iodine enhancement images were acquired 7 minutes after contrast injection.





**Supplementary Figure 2:** **Flowchart showing study population enrollment**

Patients who underwent both cardiac dual-layer spectral detector computed tomography (SDCT) and cardiac magnetic resonance (CMR) examinations were included in the validation group (Gv), which included patients with dilated cardiomyopathy (n = 23), hypertrophic cardiomyopathy (n = 4), hypertensive heart disease (n = 3), diabetic cardiomyopathy (n = 4), valvular heart disease (n = 4). There were also seven controls included.


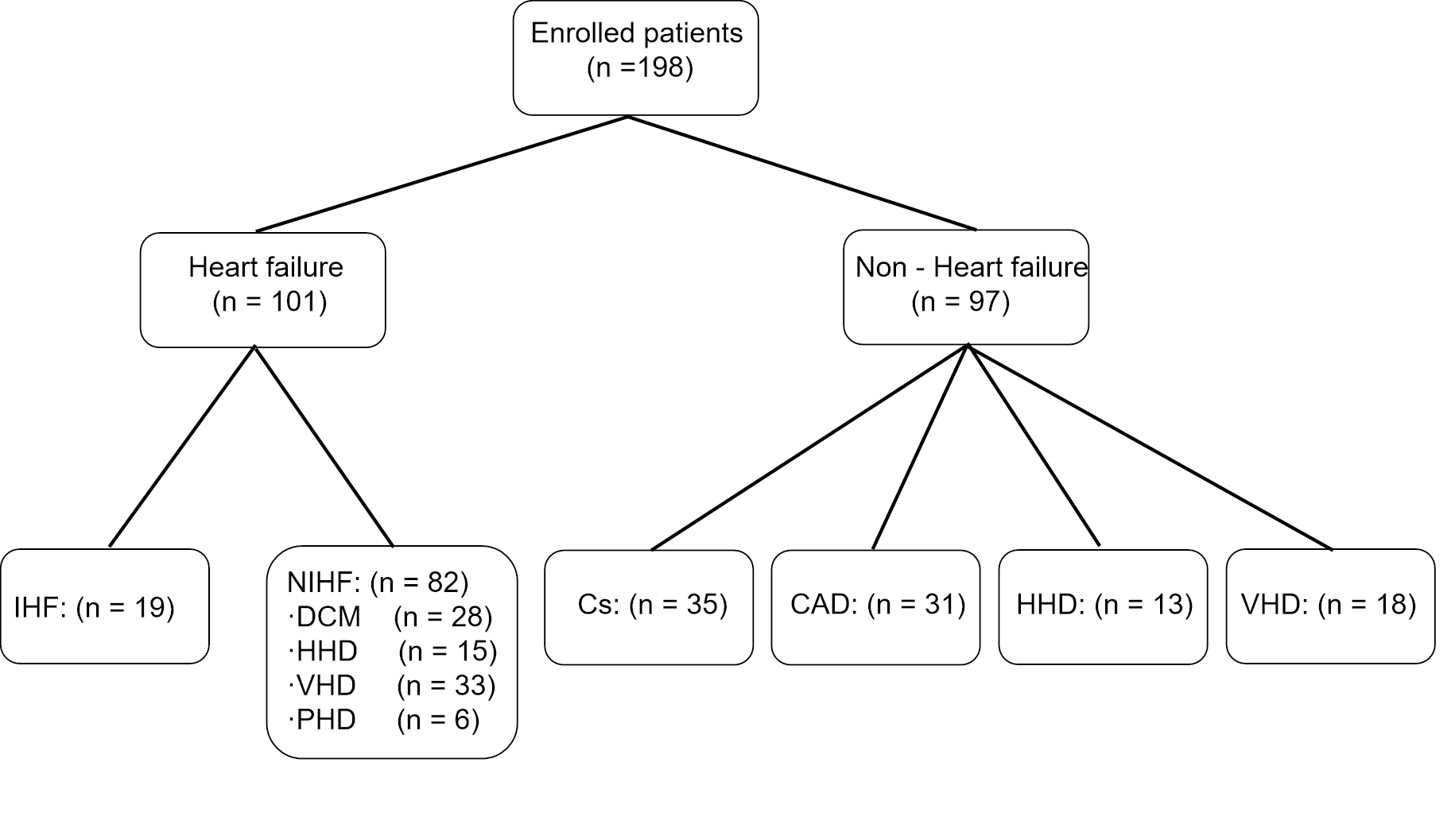


**Supplementary Figure 3:** **Selection chart of patients and controls**

IHF = Ischemic heart failure; NIHF = non-ischemic heart failure; DCM = dilated cardiomyopathy; HHD = hypertensive heart disease; VHD = valvular heart disease; PHD = pulmonary heart disease; Cs = controls; CAD = coronary artery disease
